# The Impact of Imidacloprid in Dietary Residues on Intestinal Damage and the Increased Risk of Enterotoxigenic *Escherichia coli* Infection

**DOI:** 10.3390/foods14122119

**Published:** 2025-06-17

**Authors:** Xinlei Yuan, Zihan Wang, Fang Wu, Le Cheng, Yutong Jin, Jianguo Dong, Chenyan Zheng, Yumeng Ma, Yan Jin, Bing Fang

**Affiliations:** 1 State Key Laboratory of Food Nutrition and Safety, College of Food Science and Engineering, Tianjin University of Science and Technology, Tianjin 300457, China; yuanxinlei@mail.tust.edu.cn (X.Y.); jinyan@tust.edu.cn (Y.J.); 2 Key Laboratory of Precision Nutrition and Food Quality, Department of Nutrition and Health, China Agricultural University, Beijing 100193, China; wangzihan01213@163.com (Z.W.); wufang@cau.edu.cn (F.W.); chengle@cau.edu.cn (L.C.); sy20233313727@cau.edu.cn (Y.J.); 2860101335@cau.edu.cn (J.D.); chenyan.zheng@cau.edu.cn (C.Z.); b20243311368@cau.edu.cn (Y.M.)

**Keywords:** imidacloprid, intestinal stem cell, intestinal mucosal immunity, infection, health risk

## Abstract

Pesticide residues in foods can disturb the intestinal barrier and microbiota, even at a very low dose; however, studies on direct consequences on intestinal health are still lacking. Here, we evaluated the damage of imidacloprid (IMI) to the intestine and the resulting defense against enterotoxigenic *Escherichia coli* (ETEC) in C57BL/6J mice. After 8-week exposure to 0.06 mg /kg bodyweight/day, IMI significantly damaged intestinal structure and intestinal integrity, characterized by an increased permeability to FITC-dextran and decreased mRNA expression of tight junction proteins, as well as more broken villi and lower proportions of goblet cells and paneth cells. These were related to the suppression of the self-renewal of intestinal stem cells (ISCs), as evidenced by significantly decreased Sox9+ ISCs and increased apoptosis. Furthermore, the impaired intestinal integrity in mice exposed to low doses of IMI directly increased the susceptibility to ETEC infection and even caused death. On the other hand, exposure to 0.6 mg IMI/kg bodyweight/day lead to significantly increased contents of IL-1β and TNFα both in the intestine and serum, and significantly decreased Th1 cell and IFN-γ contents in the lamina propria during the ETEC infection. Our study suggested that the intestinal damage induced by pesticide residues would significantly decrease the defense ability of the intestine, which suggests a novel perspective when evaluating the long-term effects of food contaminates on intestinal health at low doses without significant toxicological injuries.

## 1. Introduction

The intestine is the first line of defense against harmful components in the diet through the microbial barrier, chemical barrier, physical barrier, and immune barrier, which in total form the intestinal mucosal immune system [1]. The gut microbiota plays an important role in the maintenance of homeostasis, modulation of immune response, and defense against pathogenic infections in the intestinal tract [2]. As a result, several studies have focused on the changes in the composition of gut microbiota when evaluating the health risks of toxic food chemicals such as pesticide residues [3,4,5,6]. Although several studies have reported a correlation between altered gut microbiota and chronic diseases, they still do not directly reflect what would happen to the health of the body based on alterations in the gut microbiota induced by pesticides [7,8,9].

The other frequently evaluated index is gut permeability, according to the expression of tight junction proteins, including C (ZO-1), Occludin, and Claudins [10,11,12]. It was found that damaged intestinal epithelial cells can be repaired or renewed by C(ISCs) located in the crypt [13,14]. Besides maintaining intestinal homeostasis, ISCs can differentiate into goblet cells and paneth cells, which can defend against antigens and pathogenic bacteria in the diet through secreting mucins and lysozymes, respectively [13,15,16,17]. The lamina propria, beneath the intestinal epithelium, contains a series of immune cells that receive antigenic signals from dendritic cells and microfollicular cells in the epithelium and differentiate into regulatory T (Treg) cells and helper T (Th1, Th2, and Th17) cells, which perform intestinal-specific immune functions by releasing cytokines [16]. However, the existing toxicological evaluations or health risk assessments of food contaminants usually neglect the influence on the proliferation and differentiation of ISCs and immune cells in the lamina propria.

Imidacloprid (IMI) has been widely used for the control of yield-reducing pests since its introduction. In two successive comprehensive dietary studies examining the dietary exposure and health risks associated with neonicotinoid pesticides among the general population in China, researchers discovered that IMI was the most prevalent neonicotinoid pesticide in the country, presenting a detection rate exceeding 60% across all tested food samples [18]. Also, IMI is present as the most frequently detected neonicotinoid in the data published in the USDA Pesticide Data Program [19]. These findings indicate that dietary intake of agricultural products containing IMI residues represents the primary exposure route for the general population. Chen et al. revealed that IMI had the greatest detection rate in a study of serum neonicotinoid concentrations in the general population of Wuxi, Eastern China [20]. The maximum residue limit (MRL) of imidacloprid in food is influenced by a combination of factors such as the risk assessment models of different countries, agricultural production practices, and differences in dietary structures. This leads to variations in the MRL standards across different food categories and regions. Specifically, the Codex Alimentarius Commission has set the MRL standards as follows: for nut products, the limits for almonds, Brazil nuts, and macadamia nuts are all 0.01 mg/kg; for fruit products, the limit for pears is 0.5 mg/kg, 0.5 mg/kg for peaches, and 0.3 mg/kg for plums [21]. In contrast, the European Food Safety Authority has established different MRL standards, stipulating that for nut products, the limits for almonds, Brazil nuts, and macadamia nuts are 0.05 mg/kg; for fruit products, the limit for pears is 1 mg/kg, and for both peaches and plums, the limit is 1.5 mg/kg [22].

In conventional toxicology research, oral administration of IMI to mice or rats at doses of 15 or 20 mg/kg bw/day resulted in significant reductions in body weight accompanied by visible signs of toxicity [23]. Health risk assessments of IMI have been carried out, and at doses of 1.0 mg/kg bw/d, revealed that IMI disrupts insulin homeostasis and affects glucose–lipid metabolism, in addition to destroying intestinal tight junction proteins and thus increasing intestinal permeability [24,25]. However, negligible toxicity was shown in dietary exposure to neonicotinoid pesticides in children though consuming fruits and vegetables [26,27]. These results indicate that it is necessary to clarify the effects at the actual doses people are exposed to or which are claimed to be safe, and if they directly exhibit consequences induced by damaged intestinal health. Therefore, in this study, we systematically evaluated the effects of IMI on the intestinal mucosal immune system and the corresponding defense ability using enterotoxigenic *Escherichia coli* (ETEC) at low doses and high doses. This study found that IMI could damage intestinal permeability at low doses and exhibit different injury mechanisms at different doses, which demonstrates a novel perspective when evaluating the long-term effects of food contaminates on intestinal health at low doses.

## 2. Materials and Methods

### 2.1. Animals and Chemicals

All experimental procedures were approved by the Institutional Animal Care Committee as well as the Ethics Committee at China Agricultural University (AW02304202-5-1). C57BL/6J mice (8 weeks old, half male and half female) were supplied by SPF (Beijing, China) Biotechnology Co., Ltd. IMI with a purity of more than 97% provided by Weishi Regent (Wuhan, Hubei, China).

IMI (purity > 97%, Weishi Regent), dimethyl sulfoxide (DMSO, D8370, Solarbio, Beijing, China), physiological saline mixture (FS20565, Feimobio, Beijing, China), Carnoy’s solution (D1710, Solarbio, Beijing, China), ETEC strain (10667, CICC, Beijing, China), Trizol reagent (15596026, Invitrogen, Waltham, MA, USA), All-In-One 5X RT Mastermix (G492, ABM, Richmond, BC, Canada), SYBR Green (RR82LR, Takara, San Jose, CA, USA).

### 2.2. Design of the Experiment

IMI was dissolved in dimethyl sulfoxide (DMSO, Solarbio, Beijing, China) to form a stock solution, which was then diluted with 0.9% saline (Feimobio, Beijing, China) containing Tween-20. The final concentrations of DMSO and Tween-20 in the gavage solution were 0.1% and 0.5%, respectively. Fifty-four C57BL/6J mice (20.8 ± 0.4 g) were divided into three groups randomly (n = 18, half male and half female), the Con group (0 mg/kg bw/d with vehicle), the low-dose group (0.06 mg/kg bw/d), and the high-dose group (0.6 mg/kg bw/d), and all mice were gavaged daily for 8 weeks.

All animals were provided with the same standard pellet diet and had free access to food and distilled water. Throughout the experiment, mice were housed in cages within a single room maintained at 24 ± 2 °C and 40–70% relative humidity, following a 12 h light/dark cycle.

At the end of the exposure period, rats were anesthetized with diethyl ether, blood was collected from the orbital sinus, and then they were euthanized by decapitation. Blood was collected in plain tubes and allowed to stand at 37 °C for 2 h, followed by centrifugation at 5000× *g* for 20 min at 4 °C. The supernatant serum was transferred to new sterile tubes and stored at −80 °C until further analysis. During the administration of IMI, the body weight of each mouse and total food intake per group were measured weekly.

### 2.3. In Vitro Intestinal Permeability Assay

Jejunum samples 10 cm in length were isolated, injected with FITC-dextran (Aladdin, Shanghai, China) solution, and then placed in Krebs–Henseleit bicarbonate buffer for 20 min of incubation. A multi-functional microplate reader (Synergy H1, BioTek, Winooski, VT, USA) with an emission wavelength of 530 nm and an excitation wavelength of 485 nm was applied to the fluorescence degree of the buffer. This experiment was conducted under light-avoidance conditions throughout. FITC-dextran permeation per centimeter per minute was calculated to obtain permeability data [28].

### 2.4. Histopathological Examination

Intestinal tissues were immersed in Carnoy’s solution (Solarbio, Beijing, China), and then embedded in paraffin, and cut into 4 mm sections. Sections were subjected to hematoxylin–eosin (H&E) staining, alcian blue-picric acid Schiff (AB-PAS) staining, fluorescent double-labeled trichrome staining, and TUNEL staining. Then, observations were performed using a 100× upright optical microscope (M165FC, Leica Microsystems, Wetzlar, Germany). For each group, three tissue samples were used per gender, with six fields of view observed for each tissue. Quantification and statistical analysis were conducted using ImageJ software (Version 1.44C).

### 2.5. Short-Chain Fatty Acid Analysis

Contents of short-chain fatty acids (SCFAs) in feces were measured by gas chromatography (8860, Agilent, Santa Clara, CA, USA), utilizing an adapted methodology based on prior research for the subsequent quantitative assessment [29].

### 2.6. ETEC Infection

The ETEC strain 10667 (CICC, Beijing, China) was cultured in Luria–Bertani broth at 37 °C for 24 h until the logarithmic growth phase. The bacterial suspension was centrifuged to collect cells, which were then resuspended in physiological saline. Subsequently, mice were orally administered a dose of 1 × 108 CFU over two successive days. Throughout the infection period, the mice’s body weights were recorded every 12 h, while their total food consumption and fecal output were assessed every 24 h for each group. Fecal characteristics and computed disease activity index (DAI) score were also monitored.

### 2.7. ETEC Burden in Fecal Samples

The presence of ETEC in fecal samples was assessed using previously outlined methods. In summary, fresh stools were gathered, mixed with saline to create a homogenate, and subsequently plated on MacConkey agar (Merck, Darmstadt, Germany) utilizing a gradient dilution technique. After incubating for 24 h at 37 °C, the number of colony-forming units (CFUs) was determined [30].

### 2.8. Flow Cytometry Analysis of T Lymphocytes Located in Intestinal Lamina Propria

Cells from the intestinal lamina propria were isolated using established methods [31]. The cell suspensions above were enhanced with the fluorophore-conjugated antibodies: BD OptiBuildTM BUV496 Rat Anti-Mouse CD4, BD PharmingenTM PE RatAnti-Mouse IL-17A, Brilliant Violet 421TM anti-mouse CD45 Antibody, APC/Cyanine7 anti-mouse CD3 Antibody, FITC anti-mouse CD25Antibody, PE/Cyanine7 anti-mouse IFN-γ Antibody, APC anti-mouse IL-4Antibody (741050, 559502, 103134, 100222, 101908, 505826, 504106, respectively, Biolegend, Beijing, China). The cells were incubated with the antibody mixture for 30 min while kept on ice and shielded from light. Following this, they underwent fluorescence-activated cell sorting utilizing the Becton Dickinson FACSAriaTM III (BD Bioscience, San Jose, CA, USA).

### 2.9. Gene Expression Analysis

Total RNA was isolated from both the jejunum and ileum using TRIzol reagent (Invitrogen, Carlsbad, CA, USA,) in conjunction with other essential reagents. In order to synthesize complementary cDNA from the extracted RNA (2000 ng), All-In-One 5X RT Mastermix (ABM, Los Angeles, CA, USA) was utilized. Gene expression analysis was performed using a quantitative PCR instrument (Light Cycler 96, Roche Molecular Systems, Pleasanton, CA, USA) via real-time quantitative PCR, with SYBR Green (Takara, Kusatsu, Japan) serving as the detection agent, and the results were normalized against GAPDH. The primer pair sequences are shown in Table 1. Gene expression levels were measured employing the 2^−∆∆^^CT^ method.

### 2.10. Inflammatory Cytokine Measurements

The levels of Interleukin-1 beta (IL-1β) and tumor necrosis factor-α (TNF-α) in intestinal tissues and serum were evaluated using colorimetric assay kits (Multi Sciences, Hebei, China). In the end, the optical density was recorded at 450 nm with a microplate ELISA reader (Biotek, Winooski, VT, USA).

### 2.11. Statistical Analysis

Comparisons between two groups were made using unpaired two-tailed Student’s *t*-tests, and comparisons of multiple groups were made using one-way ANOVA followed by Tukey’s multiple comparisons test, all carried out with GraphPad Prism version 9.3.0 for data analysis. A *p*-value of less than 0.05 was considered significant (marked with * or #), while *p*-values below 0.01 (noted as ** or ##) indicated even higher significance. Results are expressed as the mean ± SEM for the group.

## 3. Results

### 3.1. Exposure to Low Dose of IMI Significantly Damaged Intestinal Integrity Whereas High Dose of IMI Just Induced Inflammation

**Figure 1:**
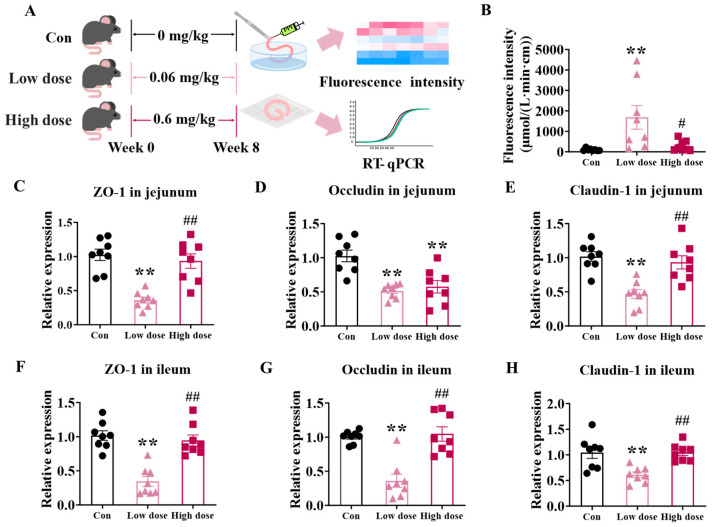
Effects of IMI exposure on intestinal epithelial integrity. (**A**) Experiment design. (**B**) Intestinal permeability measured by the permeation of FITC-dextran ex vivo. (**C**–**H**) mRNA expression of tight junction proteins in the jejunum (**C**–**E**) and ileum (**F**–**H**). Circles, triangles, and squares represent the Con, low-dose and high-dose groups. Values are presented as the mean ± SEM. *p*-values were determined using one-way ANOVA followed by Tukey’s multiple comparisons test. # *p* < 0.05, **/## *p* < 0.01 (* low dose/high dose vs. Con; # high dose vs. low dose), n = 8.

**Figure A1:**
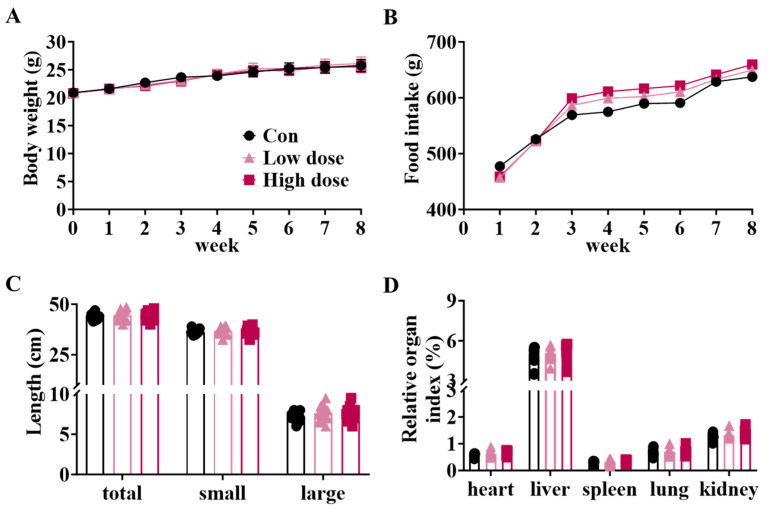
Effects of IMI exposure on body weight (**A**), food intake (**B**), intestine length (**C**), and relative organ index (**D**) of mice. Circles, triangles, and squares represent the Con, low-dose and high-dose groups. Values are expressed as the mean ± SEM of biologically independent samples. *p*-values were determined using one-way ANOVA followed by Tukey’s multiple comparisons test, n = 16.

During the exposure, mice did not exhibit toxicological signatures such as weight loss, tremor, etc., and phenotypes that related to a destroyed intestinal tract. Mice exposed to IMI at a low dose (0.06 mg/kg bodyweight/day) for 8 weeks exhibited a significant increased permeability of dextran compared with those in both the Con and high-dose groups (Figure 1B, *p* < 0.01). Further investigations of the expression of tight junction proteins in the jejunum (Figure 1C–E) and ileum (Figure 1F–H) also demonstrated a significant decrease in the levels of ZO-1, Occludin, and Claudin-1 at the low dose (*p* < 0.01). Mice exposed to a high dose (0.6 mg/kg bodyweight/day) of IMI did not show affected intestinal permeability or expression of tight junction proteins (*p* > 0.05). These results suggest that IMI damaged the integrity of the intestine specifically at the low dose. Figure A1 shows the effects of IMI exposure on body weight (Figure A1A), food intake (Figure A1B), intestine length (Figure A1C), and relative organ index (Figure A1D) of mice. Circles, triangles, and squares represent the Con, low-dose and high-dose groups.

**Figure 2:**
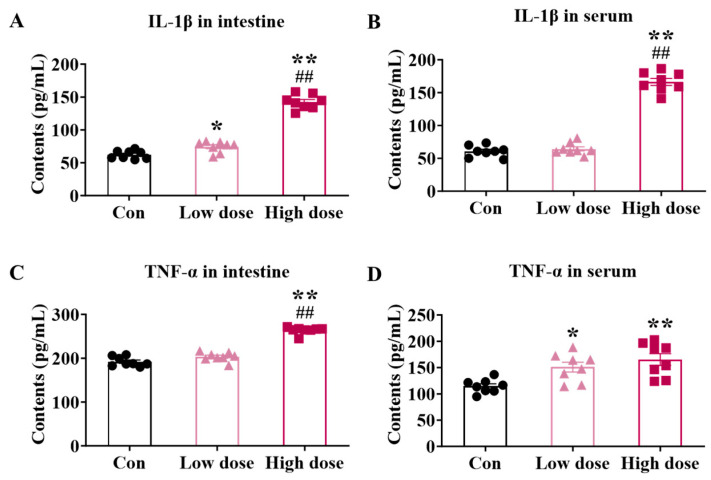
Effects of IMI exposure on intestinal and serum contents of pro-inflammatory cytokines. (**A**) IL-1β and (**B**) TNF-α contents in the intestine. (**C**) IL-1β and (**D**) TNF-α contents in the serum. Circles, triangles, and squares represent the Con, low-dose, and high-dose groups. Values are presented as the mean ± SEM. *p*-values were determined using one-way ANOVA followed by Tukey’s multiple comparisons test. * *p* < 0.05, **/## *p* < 0.01 (* low dose/high dose vs. Con; # high dose vs. low dose), n = 8.

Although the integrity of the intestine was not damaged by the high dose, the content of pro-inflammatory IL-1β and TNF-α increased significantly both in the intestine (Figure 2A,B) and serum (Figure 2C,D) compared to the Con (*p* < 0.01) and low-dose groups. There was only a significant increase in intestinal content of IL-1β (Figure 2A) and serum content of TNF-α (Figure 2D) induced by IMI at the low dose (*p* < 0.05).

### 3.2. IMI Exposure Significantly Damaged the Structure of Villi and Proliferation, but Only the Low Dose Significantly Disturbed Intestinal Stem Cells

**Figure 3:**
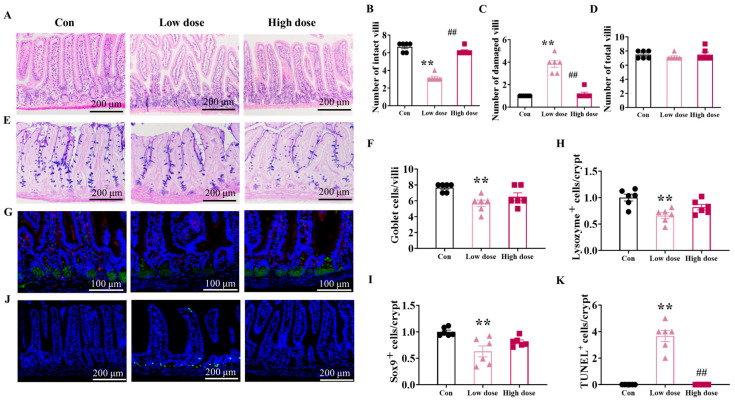
Effects of IMI exposure on intestinal structure and the associated function of intestinal stem cells. (**A**) Representative images of H&E staining of the jejunum and (**B**–**D**) the calculated numbers of intact villi (**B**), damaged villi (**C**), and total villi (**D**) in the jejunum. (**E**) Representative images of AB-PAS staining in the ileum and (**F**) the calculated number of goblet cells (labeled in purple) per villi in ileum. The scale bar in the above measurements was all set as 200 μm. (**G**) Representative images of immunofluorescence staining for Sox9+ (marker of stem cells, labeled in red) and lysozyme+ (marker of paneth cells, labeled in green), respectively (scale bar: 100 μm). (**H**,**I**) The calculated proportions of intestinal stem cells (**H**) and paneth cells (**I**) according to the fluorescence intensity. (**J**) Representative images of immunofluorescence staining for TUNEL+ cells (labeled in green, scale bar: 200 μm) and (**K**) the calculated apoptotic cells in the ileum. Circles, triangles, and squares represent the Con, low-dose, and high-dose groups. Values are presented as the mean ± SEM. *p*-values were determined using one-way ANOVA followed by Tukey’s multiple comparisons test. **/## *p* < 0.01 (* low dose/high dose vs. Con; # high dose vs. low dose), n = 6.

According to the H&E staining of the jejunum (Figure 3A), the 8-week exposure to IMI significantly damaged intestinal structure, characterized by significantly decreased numbers of intact villi along with significantly increased numbers of damaged villi, especially at the low dose (Figure 3B,C, *p* < 0.01 vs. Con and high dose). As for the composition of the differentiated cells in the ileum (Figure 3E,G), both doses of IMI decreased proportions of goblet cells (Figure 3F) and paneth cells (Figure 3H); however, the disturbance in the differentiation of intestinal stem cells was only significant at the low dose (*p* < 0.01). Furthermore, the proliferation of intestinal stem cells was inhibited by IMI according to the results of Sox9+ expression (Figure 3G,I) and apoptosis was accelerated according to TUNEL staining results (Figure 3J,K), and similarly, the effects were only significant at the low dose (*p* < 0.01).

### 3.3. IMI Exposure Decreased SCFA Production and Mucus Layer in the Colon at Both Doses

**Figure 4:**
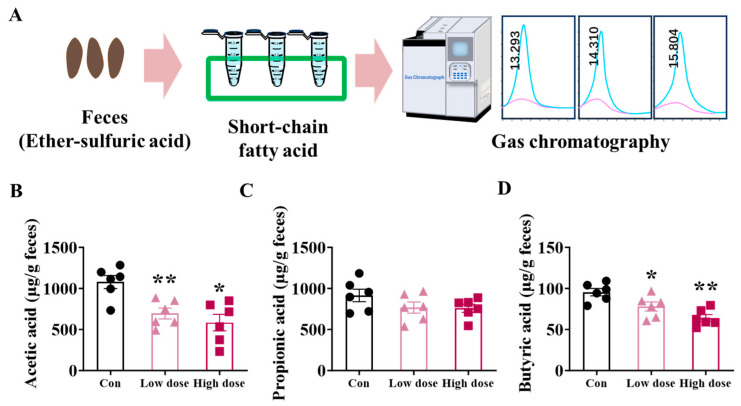
Effects of IMI exposure on contents of short-chain fatty acids (SCFAs) in the feces. (**A**) Experiment design. (**B**–**D**) Fecal contents of acetic acid (**B**), propionic acid (**C**), and butyric acid (**D**). Circles, triangles, and squares represent the Con, low-dose and high-dose groups. Values are presented as the mean ± SEM. *p*-values were determined using one-way ANOVA followed by Tukey’s multiple comparisons test. * *p* < 0.05, ** *p* < 0.01 (* low dose/high dose vs. Con), n = 6.

**Figure A2:**
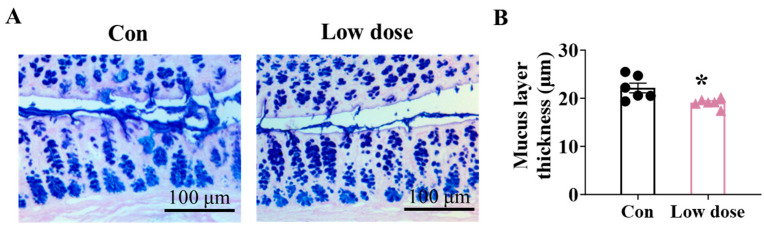
Effects of IMI exposure at the low dose on the thickness of the mucus layer of mice measured by AB-PAS staining. (**A**) Representative images of colonic AB-PAS staining (scale bar: 100 μm). (**B**) The calculated thickness of mucus layer in the colon. Circles, triangles, and squares represent the Con, low-dose, and high-dose groups. Values are expressed as the mean ± SEM of biologically independent samples. *p*-values were determined using unpaired two-tailed Student’s *t*-tests, * *p* < 0.05 (* low dose vs. Con), n = 6.

Fecal contents of acetic acid, propionic acid, and butyric acid in mice exposed to IMI at the low dose and high dose were measured at the end of the experiment (Figure 4A). IMI significantly decreased the contents of acetic acid, and butyric acid decreased in a dose-dependent way (Figure 4B, *p* < 0.05 for low dose and *p* < 0.01 for high dose). In addition, according to the AB-PAS staining of the colon (Figure A2A), IMI exposure significantly decreased the thickness of the colonic mucus layer at the low dose (Figure A2B, *p* < 0.05).

### 3.4. IMI Exposure Led to Susceptibility to ETEC Infection and Even Death in Mice of the Low Dose Group

**Figure 5:**
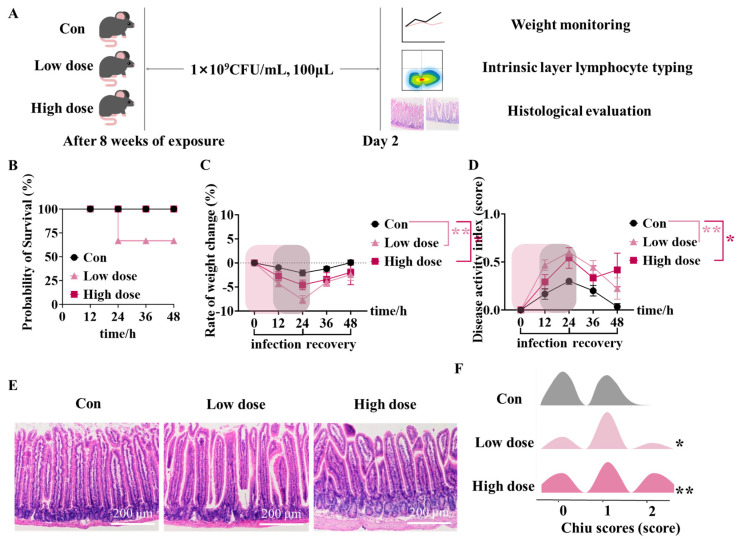
Effects of IMI exposure on the defense against ETEC infection. (**A**) Experiment design. (**B**–**D**) Survival curves (**B**), body weights (**C**), and DAI scores (**D**) of mice in each group during ETEC infection. (**E**) Representative images of H&E staining of the jejunum after ETEC infection for 72 h (scale bar: 200 μm) and (**F**) Chiu scores of the jejunum. Circles, triangles, and squares represent the Con, low-dose, and high-dose groups. Values are presented as the mean ± SEM. *p*-values were determined using one-way ANOVA followed by Tukey’s multiple comparisons test. * *p* < 0.05 (* low dose/high dose vs. Con; # high dose vs. low dose), n = 8–10. ** *p* < 0.01 (* low dose/high dose vs. Con), n = 6.

**Figure A3:**
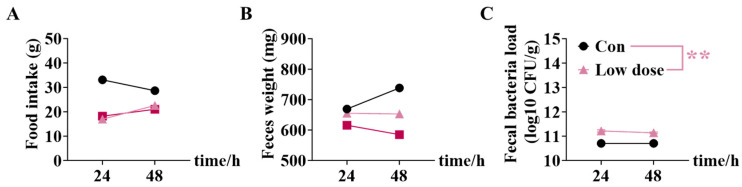
Effects of IMI exposure at the low dose on food intake (**A**), weights of feces (**B**), and fecal bacteria load (**C**) of mice during ETEC infection. Circles, triangles, and squares represent the Con, low-dose, and high-dose groups. Values are expressed as the mean ± SEM of biologically independent samples. *p*-values were determined using unpaired two-tailed Student’s *t*-tests, ** *p* < 0.01 (* Low dose vs. Con), n = 10.

Mice exposed to IMI for 8 weeks were then infected by ETEC and the death rate and intestinal structure were measured (Figure 5A). It can be seen that the survival rate of mice in the low-dose group after 24 h infection was 60%, while there were no mice dead in the Con and high-dose groups (Figure 5B). In addition, the body weights of mice in the IMI groups were significantly lower than those in the Con group (Figure 5C) and showed a slower recovery rate, especially at the low dose (*p* < 0.01), followed by the high-dose group (*p* < 0.05). DAI scores exhibited a similar result as body weight, which also suggested a more serious influence of the low dose of IMI (Figure 5D, *p* < 0.01). Furthermore, when evaluating the pathology of the intestines after infection (Figure 5E), significant vacuolation and serious injury were observed dose-dependently in the intestines of mice from the IMI groups. As illustrated in the mountain range image (Figure 5F), mice in the IMI groups had higher Chiu scores, especially in the high-dose group (*p* < 0.01). Figure A3 shows the effects of IMI exposure at the low dose on food intake (Figure A3A), weights of feces (Figure A3B), and fecal bacteria load (Figure A3C) of mice during ETEC infection.

### 3.5. Mice Survived Due to Activation of Intestinal Lamina Propria Immunity at High Dose

**Figure 6:**
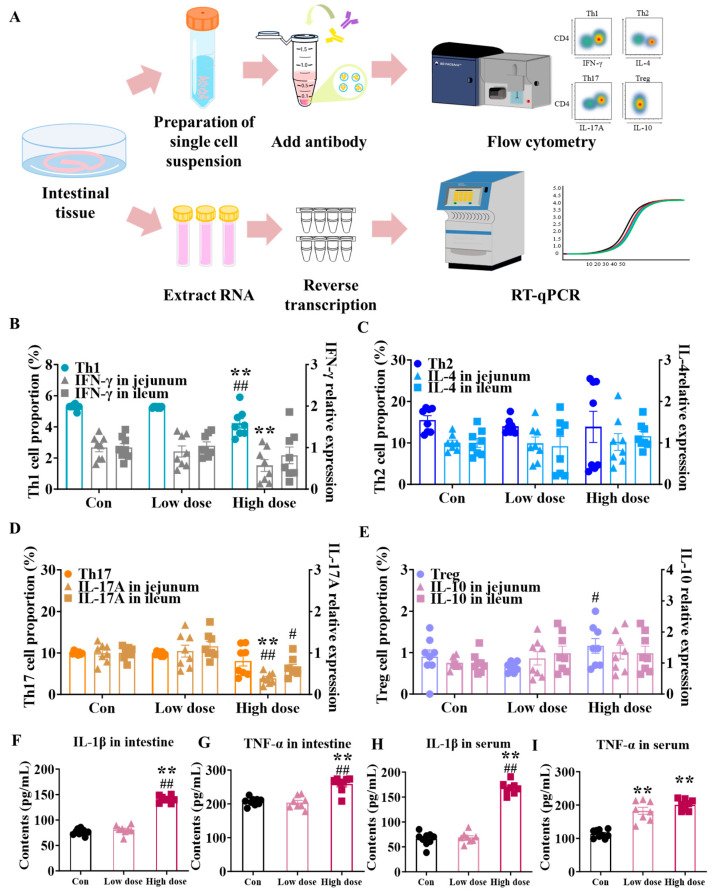
Effects of IMI exposure on T cell subtypes and the related cytokine levels in the intestinal lamina propria after ETEC infection for 72 h. (**A**) Experiment design. (**B**) Proportion of Th1 cell and mRNA expression of IFN-γ. (**C**) Proportion of Th2 cell and mRNA expression of IL-4. (**D**) Proportion of Th17 cell and mRNA expression of IL-17A. (**E**) Proportion of Treg cell and mRNA expression of IL-10. (**F**,**G**) Intestinal and (**H**,**I**) serum contents of IL-1β (**F**,**H**) and TNF-α (**G**,**I**). Circles, triangles, and squares represent the proportion of T cell subtypes and mRNA expression of cytokines in the jejunum and in the ileum, respectively. Values are presented as the mean ± SEM. *p*-values were determined using one-way ANOVA followed by Tukey’s multiple comparisons test. # *p* < 0.05, **/## *p* < 0.01 (* low dose/high dose vs. Con; # high dose vs. low dose), n = 8.

T cell subtypes in the intestinal lamina propria were measured by flow cytometry and the corresponding cytokines were also measured. As shown in Figure 6B, the percentage of Th1 cells and the expression of the relative cytokine IFN-γ in the jejunum were significantly reduced in the high -dose group (*p* < 0.01), as well as the expression of IL-17A secreted by Th17 cells (Figure 6D, *p* < 0.01), which were not changed by the low dose. In addition, IMI exposure did not significantly affect the proportions of Th2 (Figure 6C) and Treg cells (Figure 6E) and their corresponding secreted cytokines. Furthermore, the high-dose group showed significantly increased contents of pro-inflammatory IL-1β and TNF-α both in the intestine (Figure 6F,G, *p* < 0.01) and serum (Figure 6H,I, *p* < 0.01), while the low dose of IMI only significantly increased the serum content of TNF-α (Figure 6I, *p* < 0.01).

## 4. Discussion

Pesticides in the environment can enter and accumulate in the human body through the food chain and then harm human health in the long run. The intestine serves as the first line of defense against pesticides once entering the body. Although toxicologic parameters such as low doses and residue limits in food based on low doses exist, the long-term heath risks of pesticide residues even at low doses still need to be clarified. In this study, we chose IMI, a widely used neonicotinoid pesticide, to evaluate the chronic exposure-induced health risks to the intestine at low doses and the resulting changes in resistance to infection.

It is note-worthy that the 8-week exposure to IMI at the low dose specifically damaged the intestinal integrity (Figure 1B) by down-regulating the mRNA expression of genes encoded the tight junction proteins ZO-1, Claudin-1, and Occludin (Figure 1C–H), which substantially decreased the defense against ETEC infection (Figure 5). Zhao et al. also reported increased intestinal permeability and decreased tight junction protein expression in rat intestines at a dose of 0.06 mg/kg bw/d via the activation of the NF-κB-MLCK signaling pathway [25,32]. Tight junction proteins among epithelial cells establish a solid physical barrier, the proliferation and repairment of which is mediated by ISCs located in the crypts [33]. Meanwhile, ISCs can also differentiate into goblet cells and paneth cells to make up the chemical barrier of the intestine by secreting mucins and lysozyme, respectively [34]. Increased intestinal permeability was found to lead to the development of a variety of diseases, such as inflammatory bowel disease (IBD) and Crohn’s disease, and has attracted a great deal of public attention [35,36]. In this study, it was found that a low dose of IMI significantly decreased the Sox9+ ISCs and increased the apoptosis of ISCs, which may be one reason for the specific decreased intestinal defense ability, as well as the disturbed villi structure (Figure 2A–D). Although there were also significant damages to villi structure in the high-dose group, the number of Sox9+ ISCs and apoptosis of ISCs were not affected, indicating a normal repair mechanism. Secondly, significant decreases in goblet cells (Figure 3F) and paneth cells (Figure 3H) were only found in the low-dose group. Previous studies have suggested that a decline in the number of goblet cells may be related to an increase in reactive oxygen species (ROS) [37]. Therefore, the intestinal permeability (Figure 1B) and susceptibility (Figure 5) to ETEC infection in mice from a low dose did not increase.

To directly evaluate the health risk induced by disturbance to the intestinal integrity and ISC function, we assessed the defense ability of the intestine against ETEC in mice continuously exposed to IMI for 8 weeks. ETEC is a pathogen that is ubiquitous in humans and animals and produces adhesion proteins that help adhere to the epithelium of the small intestine [38]. During the infection, the mice showed mortality only in the low-dose group (Figure 5B). Moreover, we observed significantly elevated fecal load in mice at the low dose (Figure A3B). In addition, we quantified mice health status by DAI scores, including weight reduction score, blood in stool score, and fecal consistency score [39]. In concrete terms, worse health in mice correlates with higher DAI scores, and mice in the low-dose group had the highest DAI scores and the slowest recovery within 0–36h.

Different from the results in the low-dose group, at the high dose, we observed an obvious inflammation characterized by significant increases in the levels of pro-inflammatory factors both in the intestine and serum (Figure 2). Furthermore, a high dose of IMI specifically activated intestinal mucosal immunity instead of the mechanical and chemical barrier, which decreased the proportion of Th1 cells and increased Treg cells in the lamina propria (Figure 6A,E) as well as the cytokines (Figure 6A,D). T cells are crucial in the intestinal lamina propria by secreting cytokines that modulate gut-specific immunity, and in the context of IBD, Th1 cells are involved in the development and progression of intestinal inflammation by secreting factors such as IFN-γ [40,41,42,43]. Previous studies about the relief of DSS-induced colitis were associated with decreased levels of Th1 cells and their cytokines [42,44]. SCFAs also regulated immune cell differentiation, and it was reported that the high-dose group showed such an intense immune response because of the association of reduced levels of butyric acid in the feces (Figure 4). Meanwhile, it was reported that IBD patients have reduced levels of butyric acid in their feces, accompanied by elevated levels of IL-1β and TNF-α, suggesting that the mice in the high-dose group were at high risk of developing enterocolitis [45,46,47,48,49]. We used the Chiu score to assess the extent of damage to intestinal morphology by IMI in mice infected by ETEC, with higher scores representing more severe damage, and the highest scores were obtained at the high dose (Figure 5E,F) although there was no death [50,51,52]. Based on this, we speculated that the targets of IMI were different under different doses, and low doses specifically target the epithelial cells and ISCs whereas relatively high doses trigger the inflammation response. Consequently, low-dose exposure of IMI may induce a serious and immediate reaction while a higher dose may induce a long-term risk related to chronic inflammation.

## 5. Conclusions

In this study, the low dose of IMI increased intestinal permeability by disrupting the integrity of the intestinal barrier through inhibition of stem cell proliferation and differentiation and enhancement of apoptosis, resulting in susceptibility to infection. The high-dose group showed induced chronic inflammation and activated mucosal immune responses after infection. The results suggest that exposure to IMI displays different phenotypes depending on the dose and adversely affects intestinal health through multiple mechanisms. It is worth noting that the observed effects (including barrier disruption, inflammation induction, and immune response activation) were consistent between male and female individuals, with no significant gender differences detected.

## Figures and Tables

**Table 1:** The sequences of primers used in this study.

Gene Name	Forward (5′−>3′)	Reverse (5′−>3′)
GAPDH	CATCACTGCCACCCAGAAGACTG	ATGCCAGTGAGCTTCCCGTTCAG
ZO-1	GTTGGTACGGTGCCCTGAAAGA	GCTGACAGGTAGGACAGACGAT
Occludin	TGGCAAGCGATCATACCCAGAG	CTGCCTGAAGTCATCCACACTC
Claudin-1	AGATACAGTGCAAAGTCTTCGA	CAGGATGCCAATTACCATCAAG
IFN-γ	CAGCAACAGCAAGGCGAAAAAGG	TTTCCGCTTCCTGAGGCTGGAT
IL-10	CGGGAAGACAATAACTGCACCC	CGGTTAGCAGTATGTTGTCCAGC
IL-4tt	ATCATCGGCATTTTGAACGAGGTC	ACCTTGGAAGCCCTACAGACGA
IL-17A	CAGACTACCTCAACCGTTCCAC	TCCAGCTTTCCCTCCGCATTGA

## Data Availability

The data presented in this study are available on request from the corresponding author due to the data that has been used is confidential.

## Abbreviations

The following abbreviations are used in this manuscript:

IMI	Imidacloprid
ETEC	*Escherichia coli*
ZO-1	Zonula occludens-1
ISCs	Intestinal stem cells
Treg	Regulatory T
AB-PAS	Alcian blue-picric acid Schiff
H&E	Hematoxylin–eosin
SCFAs	Short-chain fatty acids
DAI	Disease activity index
CFUs	Colony-forming units
IL-1β	Interleukin-1 beta
TNF-α	Tumor necrosis factor-α
ROS	Reactive oxygen species
IBD	Inflammatory bowel disease
